# Influence of Charge, Hydrophobicity, and Size on Vitreous Pharmacokinetics of Large Molecules

**DOI:** 10.1167/tvst.8.6.1

**Published:** 2019-11-01

**Authors:** Susan R. Crowell, Kathryn Wang, Amin Famili, Whitney Shatz, Kelly M. Loyet, Vincent Chang, Yanqiu Liu, Saileta Prabhu, Amrita V. Kamath, Robert F. Kelley

**Affiliations:** 1 Preclinical and Translational Pharmacokinetics and Pharmacodynamics, Genentech, South San Francisco, CA, USA; 2 Drug Delivery, Genentech, South San Francisco, CA, USA; 3 Protein Chemistry, Genentech, South San Francisco, CA, USA; 4 Biochemical and Cellular Pharmacology, Genentech, South San Francisco, CA, USA; 5 Bioanalytical Sciences, Genentech, South San Francisco, CA, USA

**Keywords:** ocular pharmacokinetics, hydrodynamic radius, charge, hydrophobicity

## Abstract

**Purpose:**

Development of therapeutics for retinal disease with improved durability is hampered by inadequate understanding of pharmacokinetic (PK) drivers following intravitreal injection. Previous work shows that hydrodynamic radius is correlated with vitreal half-life over the range of 3 to 7 nm, and that charge and hydrophobicity influence systemic clearance. Better understanding the molecular attributes affecting vitreal elimination half-life enables improved design of therapeutics and enhances clinical translatability.

**Methods:**

Impacts of charge and hydrophobicity on vitreal PK in the rabbit were systematically assessed using antibody and antibody fragment (Fab) variant series, including ranibizumab, altered through amino acid changes in hypervariable regions of the light chain. The impact of molecule size on vitreal PK was assessed in the rabbit, nonhuman primate, and human for a range of molecules (1–45 nm, net charge −1324 to +22.9 in rabbit), including published and internal data.

**Results:**

No correlation was observed between vitreal PK and charge or hydrophobicity. Equivalent rabbit vitreal PK was observed for ranibizumab and its variants with isoelectric points (pI) in the range of 6.8 to 10.2, and hydrophobicities of the variable domain unit (FvHI) between 1009 and 1296; additional variant series had vitreal PK similarly unaffected by pI (5.4–10.2) and FvHI (1004–1358). Strong correlations were observed between vitreal half-life and hydrodynamic radius for preclinical species (*R*^2^ = 0.8794–0.9366).

**Conclusions:**

Diffusive properties of soluble large molecules, as quantified by hydrodynamic radius, make a key contribution to vitreal elimination, whereas differences in charge or hydrophobicity make minor or negligible contributions.

**Translational Relevance:**

These results support estimation of vitreal elimination rates based on molecular size in relevant preclinical species and humans.

## Introduction

Age-related macular degeneration (AMD) and diabetic retinopathy (DR) are leading causes of vision loss in aging populations, expected to cause blindness or moderate to severe vision loss in more than 14 million people by 2020.^1^ Current therapies for retinal disease typically are administered via intravitreal injection (ITV), with maximum clinical benefit requiring frequent patient monitoring and monthly or bimonthly injections in many patients. However, research has shown that patient compliance declines over time, with reduced treatment despite worsening visual outcomes.^2^–^4^ Research is focused heavily on development of longer-acting therapeutics to relieve patient burden, and to increase compliance and clinical benefit.

For systemically administered large molecule therapies, distribution and elimination are driven by processes, including paracellular diffusion, convection, and pinocytosis.^5^ Convective processes typically have a greater role than diffusion, given the large size and high polarity of protein therapeutics. Previous efforts have illustrated that charge, hydrophobicity, and FcRn recycling influence systemic clearance rates for protein therapeutics,^6^,^7^ and that these properties may be manipulated to generate therapies with favorable systemic pharmacokinetic (PK) behavior.^8^–^10^ For large molecules, such as monoclonal antibodies (Mabs) or antibody fragments (Fabs), the impact of size on systemic clearance rates typically is considered primarily with respect to the “renal filtration cut off” wherein no or very limited renal clearance occurs for molecules larger than albumin (68 kDa, ∼80 × 80 × 30 Å).^11^

Following ITV, drug distributes within the vitreous humor, into aqueous humor and retina, and ultimately the systemic circulation.^12^ The distribution of drugs following ITV is understood to be determined by factors including diffusion and convection,^13^,^14^ as well as membrane permeability (reviewed previously^12^). FcRn recycling has not been observed to significantly affect ocular PK.^15^–^17^ While some studies have suggested that the size difference between Fabs (∼50 kDa) and Mabs (∼150 kDa) did not significantly affect vitreous clearance rates in rabbit,^15^ analyses of broader size distributions have demonstrated some correlation between ocular elimination and molecular weight^12^,^18^,^19^ and diffusive properties.^20^ Though vitreous humor, comprised primarily of water and a complex network of collagen and hyaluronan, is a highly charged aqueous environment, little research has focused on the impact of charge or hydrophobicity on ocular PK in vivo.

We assessed the contribution of electrostatic charge, hydrophobicity, and molecule size to vitreal PK for a large panel of soluble large molecules administered via ITV in rabbits, nonhuman primates (NHP), and humans.

## Materials and Methods

### Description of Molecules Assessed

We used a combination of prospective rabbit PK studies of engineered charge and hydrophobicity variants, and retrospective analysis of available internal and published PK data in rabbits, NHP, and humans. A summary of the 51 molecules assessed in this study, including relevant molecular and PK properties, can be found in Supplementary Table S1.

Of the 51 molecules considered in this study, 33 were proteins, including three peptides (TA_19–TA_21), 17 antibody fragments (Fabs, TA_1–TA_15 and TA_23–TA_24), and nine full length IgGs (Mabs, TA_16–TA_18 and TA_28–TA_33); the remaining proteins included a single chain variable domain fragment (Brolucizumab, TA_22), albumin (TA_25), an Fc fusion (Aflibercept, TA_26), and a F(ab')2 (TA_27). Of the remaining 18 molecules, two were polymers (TA_34 and TA_35), three were fluorescein isothiocyanate (FITC)–dextrans (TA_36–TA_38), and 12 were protein-polymer conjugates (TA_39–TA_51).

In addition to the designed variants of ranibizumab discussed below, four more variant series (VS_2–VS_5; TA_6–TA_18) were identified among internal molecules, wherein minor sequence modifications were made during the normal course of drug development, resulting in alteration of charge and/or hydrophobicity. A broader range of molecules were included in the assessment of the relationship between molecular size and ocular PK.

### Production and Characterization of Designed Variants of Ranibizumab

To systematically evaluate the impact of charge and hydrophobicity on vitreal clearance, the sequence of ranibizumab (TA_1) was systematically varied through protein engineering, resulting in four ranibizumab variants (TA_2–TA_5). Examination of the high resolution crystal structure of the ranibizumab:vascular endothelial growth factor (VEGF) complex^21^ suggested that two complementarity determining regions (CDRs), CDR-1 (CDR-L1) and CDR-2 (CDR-L2) of the light chain, are nonantigen-contacting regions in ranibizumab. This is consistent with results of mutagenesis studies showing that alanine mutants of individual residues in these two CDRs do not perturb VEGF-binding affinity.^22^ To minimize structure and conformational alterations due to introduction of substitutions, and to ensure the variants have sufficient antigen-binding affinity for detection using assays used in PK studies, mutations were introduced only at solvent-exposed sites in CDR-L1 and CDR-L2. Sequence changes introduced in charge and hydrophobic variants are summarized in Table 1.

**Table 1:** CDR-L1 and CDR-L2 Sequences of Designed Ranibizumab Variants

Fab	CDR-L1	CDR-L2	pI	Fv HI
Ranibizumab WT	SASQDISNYLN	FTSSLHS	8.1	1212
RBZ–3	SASQDISNYLN	DTSDLES	6.8	1163
RBZ+7	RARQGIRNYLN	KTSRRHS	10.2	1009
RBZ-HI low	QASQDISNSLN	STSNLHS	8.9	1165
RBZ-HI high	SVSQVISSWLA	FASSLQT	9.1	1296

Mutations were introduced into the ranibizumab expression plasmid (Y0317) by site-directed mutagenesis using the QuikChangeII (Agilent Technologies, Santa Clara, CA) mutagenesis kit following the protocol supplied with the kit. Oligonucleotide primers specifying the required codon changes were synthesized by the Genentech (San Francisco, CA) oligonucleotide synthesis lab. Plasmids with designed changes were identified and confirmed by DNA sequencing at Genentech. Fabs were purified from cell paste from 10 L fermentation runs of *Escherichia coli* cells transformed with these plasmids. Cell paste was suspended in extraction buffer and homogenized using a microfluidizer. Fabs were captured by immunoaffinity chromatography on Protein G- Sepharose with elution buffer of 0.1 M acetic Acid at pH 2.75. The low pH eluate was buffer exchanged into 25 mM NaOAc at pH 5.0 and further purified by cation exchange chromatography on a Hitrap SP HP prepacked column. Identities of the purified proteins were confirmed by mass spectroscopy and the pooled fractions were concentrated to approximately 10 mg/mL, and exchanged into phosphate buffered saline (PBS) buffer, via diafiltration.

Surface plasmon resonance (SPR) measurements on a Biacore T200 instrument (GE Healthcare, Chicago, IL) were used to confirm high affinity binding (*K_D_* < 5 nM) of these Fabs to immobilized VEGF, sufficient for use of VEGF-binding enzyme-linked immunosorbent assay (ELISA) for determination of drug concentrations in PK studies.

### Characterization of Charge, Hydrophobicity, and Molecular Size

Isoelectric point (pI) values were determined for designed ranibizumab variants and several additional Fab and IgG variant series (TA_1–TA_18) using imaged capillary isoelectric focusing as described in by Li et al.^23^ Net charge, estimated based on protein sequence and chemical structures as appropriate, was calculated using the Henderson-Hasselbalch equation, the number of ionizable residues, and by using fixed pKas for the ionizable residues.

Hydrophobicity of the antibody Fv domains for TA_1–TA_18 was calculated according to the empirical model (using the Eisenberg scale) described by Bumbaca Yadav et al.^7^ Elution time on a 4.6 × 100 mm Thermo MabPacHIC-10 column also was determined for selected antibody Fabs (Supplementary Table S1). Mobile phase A consisted of 2.0 M ammonium sulfate, 100 mM sodium phosphate pH 7.0, and buffer B was 100 mM sodium phosphate pH 7.0. The column was equilibrated in 100% A at a flow rate of 1.0 mL/min and temperature of 25°C. Injections of 10 μg protein were performed. Proteins were eluted with a linear gradient over 29 minutes of 0% to 100% buffer B and detected by absorbance at 214 nm. Hydrodynamic radius (R_H_) of proteins and protein conjugated materials were determined as described previously^20^ using size exclusion chromatography with quasielastic light scattering detection (SEC-QELS).

### In Vivo PK Studies

PK data were determined following ITV administration for designed ranibizumab charge variants in New Zealand white rabbits, and for retrospectively assessed test articles in New Zealand white Rabbits and/or cynomolgus monkeys as noted in Supplementary Table S1. All animal studies were conducted in accordance with ethical standards of the Genentech institutional animal care and use committee guidelines and in agreement with the ARVO Statement for the Use of Animals in Ophthalmic and Vision Research. Animal studies were conducted at the laboratory animal resource facility at Genentech, or at Assessment and Accreditation of Laboratory Animal Care accredited contract research organizations.

In all Genentech conducted animal studies included herein, test articles were administered by a board certified veterinary ophthalmologist. Test articles typically were formulated in sterile PBS (pH 7.4) or formulation buffer (pH 5.5, 10 mM His-HCL, 10% trehalose, 0.01% polysorbate20), typically at protein concentrations of 10 mg/mL such that a 50 uL injection delivered 0.5 mg/eye dose. In the case of radiolabeled test articles, Iodine-125 (I-125) was purchased from PerkinElmer Life and Analytical Sciences, Inc. (Waltham, MA), and applied through indirect labeling of lysine residues as described previously.^24^

Following administration, vitreous humor was harvested at terminal collections, and aqueous humor was harvested in-life via aspiration, or terminally. Test article concentrations were determined by ELISA, mass spectrometry, and/or direct analysis of radioactivity via gamma counter for I-125 labeled test articles. In ELISA for ranibizumab variants (TA_1-5), standards, controls, or samples were captured with recombinant human VEGF (Genentech) coated on Nunc MaxiSorp clear 96-well plate (Thermo Fisher Scientific, Waltham, MA) and detected by HRP AffiniPure F(ab')2 fragment goat anti-human IgG (Jackson ImmunoResearch Laboratories, West Grove, PA). After color development with tetramethylbenzidine (TMB), optical density was read at 450 nM (reference 650 nM) on a SpectraMax 190 plate reader (Molecular Devices, San Jose, CA). Data were analyzed using Softmax Pro v6.5.1 (Molecular Devices) using a four-parameter logistic fit with 1/y^2 weighting. For other test articles where ELISA was used to determine concentrations, ELISA consisted of capture with coated antigen followed by detection with antibody to analyte or capturing and detecting with two different antibodies to analyte; additional details on detection methods are summarized alongside molecule information in Supplementary Table S1.

### PK Data and Analyses

PK parameters were determined by noncompartmental analysis with nominal time and dose except where noted (Phoenix WinNonlin, Certara, Inc., Mountain View, CA).

Additional PK data in rabbits, NHP, and humans were taken from published literature (Supplementary Table S1). PK data in humans generally are restricted to analysis of aqueous or serum, with vitreous PK inferred based on modeling as described by Xu et al.^25^

Linear and log–log regression models were fitted to data describing molecular attributes and PK (Prism; GraphPad Software, Inc., La Jolla, CA).

## Results

The correlation between calculated hydrophobicity index of the variable domain unit (FvHI) and elution time of a subset of molecules (*n* = 12) on a hydrophobic interaction column was strong (*R*^2^ = 0.8826; Supplementary Fig. S1), suggesting that sequence-based calculations are valid for ranking relative hydrophobicity. A comparator for electrostatic charge was provided by measurements of the pI of the molecules. Molecular attributes and PK properties for antibody and Fab variant series can be found in Supplementary Table S1. The pI across molecular variants series ranged from 5.4 to 10.2; ranibizumab variants had the broadest range of pI within a single molecule series (6.8–10.2). Calculated FvHI across protein variant series ranged from 1004 to 1358; ranibizumab variants ranged from 1009 to 1296. Calculated net charge across protein variant series ranged from −16 to +16.9; ranibizumab variants ranged from −0.8 to +9.3, and the entirety of molecules in this study ranged from −1324 to +22.9.

**Figure 1:**
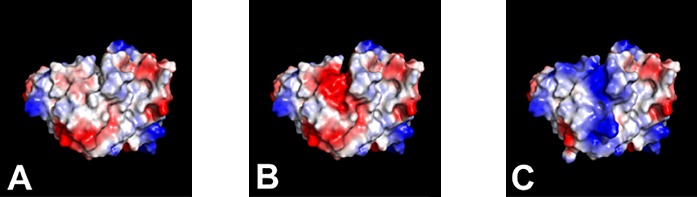
Charge surfaces modeled onto the structure of ranibizumab for ranibizumab WT (A, TA_1), RBZ_var1 (B, TA_2); and RBZ_var2 (C, TA_3). Nonantigen contact CDRs L1 and L2 were targeted for mutagenesis, with mutations introduced at solvent exposed sites. Clustered charge variants with relative charge versus WT of −3 (B) and +7 (C) were chosen for PK evaluation. Positive charge is shown in blue, negative in red, and neutral in white.

For the designed variants of ranibizumab, it was desirable to maintain VEGF-binding such that antigen-binding could be used as a detection method for study samples. Since light chain CDRs 1 and 2 are not contacting regions between ranibizumab and VEGF in the co-crystal structure,^21^ amino acid substitutions were introduced in these regions (Table 1) to produce variants that retained high-affinity binding to VEGF. As shown for the electrostatic charge surface (Fig. 1), this approach produced clustered changes in charge or hydrophobicity. Similarly, variant series 2 and 5 (TA_6-TA_8, TA_16-TA_18, Supplementary Table S1) differed by changes in one or two CDRs altering a surface patch. In contrast, variant series 3 and 4 (TA_9-TA_15) involved changes in CDR and variable domain framework residues such that the charge differences were more diffuse.

**Figure 2:**
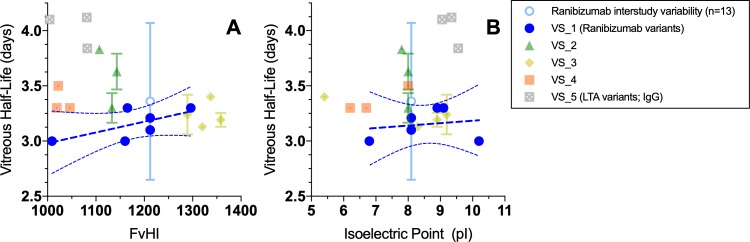
Relationship between vitreous half-life and (a) hydrophobicity or (b) isoelectric point for ITV administered fab and IgG variant series in New Zealand white rabbits. Linear regressions with 95% confidence intervals are shown in dark blue for ranibizumab series, with R^2^ = 0.4485 (A) and R^2^ = 0.0333 (B). Note that standard deviation across 13 studies of ITV ranibizumab, shown in light blue, exceeds the magnitude of distribution for any variant series shown.

There was no significant trend between variation in FvHI or pI and vitreal half-life in rabbits within any or across all molecule variant series assessed (Fig. 2). Notably, the range of half-lives observed was within the interstudy variability determined for ranibizumab vitreous half-life in rabbits across 13 internal PK studies (3.4 ± 0.71 days, Supplementary Table S2). Also, no strong trends were observed between FvHI or pI and vitreal clearance or volume of distribution (Supplementary Fig. S2).

**Figure 3:**
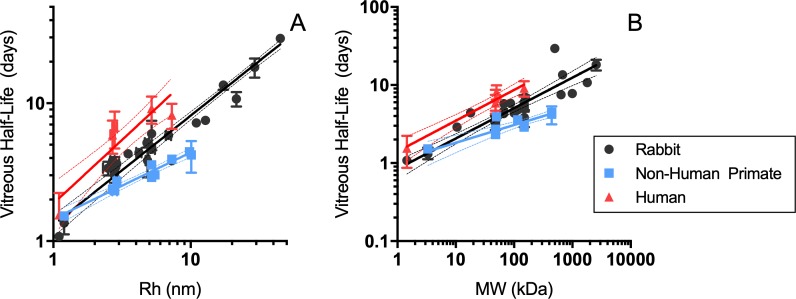
Relationships between vitreous half-life and (a) hydrodynamic radius (R_H_, nm) or (b) molecular weight (MW, kDa) in rabbit (black), NHP (blue), and humans (red). Log–log regressions with 95% confidence intervals are shown for half-life and R_H_ (A; rabbit, R^2^ = 0.9362; NHP, R^2^ = 0.9238; human, R^2^ = 0.7730) and MW (B; rabbit, R^2^ = 0.7216; NHP, R^2^ = 0.7774; human, R^2^ = 0.8618).

The relationship between molecular size and vitreal half-life in rabbits, NHP, and humans can be found in Figure 3. Data suggest strong correlation between hydrodynamic radius and vitreous half-life across molecules ranging from 1.1 to 45 nm in rabbit (*R*^2^ = 0.9366) and 1.2 to 10.1 nm in NHP (*R*^2^ = 0.9238); limited human data support a moderate correlation for molecules ranging from 1.1 to 7.3 nm (*R*^2^ = 0.773). Compared to hydrodynamic radius, molecular weight was somewhat less strongly correlated with vitreal half-life in rabbits (1.45–2570 kDa, *R*^2^ = 0.7183) and NHP (3.3–440 kDa, *R*^2^ = 0.7774), whereas the correlation in humans was stronger (1.45–148 kDa, *R*^2^ = 0.8618). Correlations between clearance and hydrodynamic radius or molecular weight generally were weaker than for half-life; no trends were observed between molecular size and volume of distribution (Supplementary Fig. S3).

## Discussion

Vitreal PK does not appear to be measurably affected by variation in hydrophobicity or pI for any of five Fab or IgG variant series evaluated, nor across all Fabs assessed. Variation in molecule charge did not produce significant alteration of vitreal half-life independent of whether engineering produced localized (variant series 1, 2, 5) or more distributed (variant series 3, 4) changes in electrostatic surface. In contrast, when systemically administered, increased net positive charge, such as for variants of anti-lymphotoxin α (anti-LTα, VS_5; TA_16-18), has been observed to result in significantly faster nonspecific clearance in rodents and NHP.^7^ While the precise mechanisms for increased systemic clearance of more positively charged antibodies are not proven, hypotheses center on enhanced rates of pinocytosis due to electrostatic interactions with negatively charged components of endothelial cells, such as glycosaminoglycans and proteoglycans,^9^,^26^,^27^ or prolonged association with FcRn.^28^

The vitreous humor is a highly aqueous and nearly acellular matrix enclosed by the hyaloid membrane, and surrounded by cellular tissues (lens, ciliary body, retina, choroid, and sclera). Further, the vitreous contains a complex network of collagen fibrils, as well as highly negatively charged hyaluronic acid; these components form a mesh with pore sizes estimated at approximately 550 nm.^29^ Thus, manipulation of charge and hydrophobicity present possible levers for affecting vitreal elimination rate, through altering rates of pinocytosis into surrounding cellular tissues, or via electrostatic interactions with the vitreous environment leading to retention or repulsion. Others have observed in in vitro and ex vivo systems such that manipulation of charge and/or hydrophobicity may affect particle and molecular diffusion in vitreous, particularly for strongly cationic molecules.^29^–^33^ Based on experiments conducted in ex vivo ovine vitreous, Kasdorf et al.^33^ propose the existence of a molecular charge threshold (i.e., net charge per molecule, as opposed to charge density) above which diffusion through vitreous is inhibited, despite molecular size much smaller than the vitreous mesh. Here, assessment of the isolated impacts of charge (pI) and hydrophobicity (FvHI) were initially limited to soluble proteins with molecular size approximately 2.5 to 5.5 nm, using engineered variants as a means of experimental control. Due to the broad distribution of size and disparate molecular properties of the entire range of materials in this study, the full breadth of test articles can best be compared based on net charge (calculated from protein sequences and chemical structures; Supplementary Table S1). Our results for cationic antibodies, for example, anti-LTα_v+3 (TA_18, +22.9), anti-LTα_WT (TA_16, +16.9), and Rituximab (TA_31, +16.9), for which vitreal elimination rates in rabbits were not significantly different than for other less cationic antibodies, suggested that a charge threshold as proposed by Kasdorf et al.^33^ may not be entirely independent of charge density (Supplementary Fig. S4). Charge threshold also may vary between species; notably, rabbit vitreous has lower concentrations of hyaluronan than does that of sheep or humans,^33^,^34^ which could dictate a higher positive charge threshold for suppression of diffusion in rabbits. Alternatively, it is also plausible that in vivo, electrostatic, and/or hydrophobic interaction with vitreous constituents could have a role in spatial distribution within vitreous humor without significantly influencing overall rates of vitreal elimination through inhibited diffusion or enhanced pinocytosis. The impacts of charge and hydrophobicity also could be more apparent in tissue matrices surrounding the vitreous environment, consistent with the established effects of charge on tissue distribution and clearance for protein therapeutics.^9^ However, current methods for assessment of impact to retinal distribution are not sufficiently quantitative or precise for application to in vivo PK, due to the technical challenges of isolating the tissue without contamination from adjacent vitreous or choroid.

Molecule size and vitreal elimination are correlated strongly in rabbits, NHP, and in the limited available data, humans. The R_H_ quantifies the diffusive properties of a molecule, and is defined as the radius of an equivalent sphere diffusing at the same rate as a given molecule in solution, as measured experimentally by light scattering techniques. The conclusion that diffusion is a critical driver of vitreous PK is reinforced by the observation that vitreal elimination tended to be more strongly correlated with R_H_ than molecular weight. R_H_ is not strictly proportional to molecular weight, but rather a holistic descriptor of volume and spatial properties, such as rigidity and molecular interactions, which can vary dramatically for molecules with such disparate physical chemical properties as those assessed here.

While these data strongly supported the role of diffusion in dictating vitreal elimination rates, the observed interspecies differences in vitreal half-lives suggested that other factors must be contributing. That is, while physiologic vitreous volume (and thus, diffusional distance) is smaller in rabbits than in NHP, observed vitreous half-lives are longer in rabbits for a given molecule, or for molecules of a given hydrodynamic radius. Thus, based on the observed relationships presented in Figure 3A, to achieve a 2-fold increase in vitreal half-life over that of a Fab (R_H_ ∼ 2.7 nm; t_1/2_ ∼ 3.4 days) in rabbits, a molecule with R_H_ of approximately 8 nm (6.7–9.7 nm) would suffice, whereas for a cyno (Fab t_1/2_ ∼ 2.3 days), a molecule with R_H_ of approximately 11 nm (8.2–15.5 nm) would be required. Several mechanistic or semimechanistic models attempt to describe ocular PKs based on anatomic, physiologic, and physicochemical factors, thus providing a foundation for interspecies translation. In the anatomic computational fluid dynamic model described by Missel,^14^ the predicted rates of elimination for a given molecule are aligned with interspecies differences in diffusional distance (i.e., longer half-lives in larger eyes) when intraocular pressure (IOP) was assumed to be the same regardless of species; however, small changes in IOP within typically observed ranges elicited significant changes in predicted rates of elimination. Several semi-mechanistic models using approaches driven by simplified ocular geometries and molecular diffusivity, while not yet applied to translation across multiple preclinical species and human, would be expected to yield predictions similarly aligned with differences in ocular anatomy (i.e., diffusional distance),^19^,^35^,^36^ and thus, at odds with the observed data we have presented.

In addition to the predicted sensitivity of vitreal elimination to IOP described by Missel,^14^ other anatomic and physiologic features could explain the observed discordance between diffusional distance and observed species-specific PK. Species differences in ocular matrix composition, pore size, and viscosity all could impact the overall rate of vitreous clearance. While the composition and nature of vitreous humor has been the subject of considerable investigation,^13^,^37^–^40^ there is no consensus on whether or how these factors influence drug distribution, nor are there robust data on interspecies differences in many aspects of vitreous composition. Indeed, while rabbit vitreous frequently is stated to be “more viscous” than that of other species (human, monkey), a conclusion that gross physical appearances of dissected vitreous supports, there is considerable debate in the field regarding how to most appropriately characterize the rheologic and mechanical properties of the vitreous.^13^,^37^,^38^,^41^,^42^ In existing ocular models, vitreous humor has been described alternately as a porous medium (e.g., Missel^14^), or a homogeneous viscous fluid (e.g., Schmitt^19^). Additionally, the possibility of entanglement or interaction with the network of collagen and hyaluronan in vitreous has been raised,^29^,^30^,^33^ but evidence of this phenomenon in vivo has not been reported for soluble materials, and interspecies differences in vitreous pore size have not been well described.

If the primary routes of egress from the vitreous humor are assumed to be through the anterior chamber and retina,^12^,^43^ differences in these interfaces and adjacent matrices could contribute to observed interspecies differences in vitreal PK. Species differences in the composition, thickness, and porosity of retinal layers, and in retinal vascularization and vascular permeability, have been noted with respect to their potential impact on drug distribution.^12^ In fact, molecular weight and particle size have been observed to affect retinal permeability and distribution ex vivo in bovine eyes.^44^,^45^ While for soluble large molecules, the contribution of the retinal pathway to overall vitreal clearance is thought to be relatively low, based on contribution of the retinal pigmented epithelium (RPE) to vitreal clearance calculated from surface area of rabbit RPE and permeabilities measured in bovine RPE (< 20%^12^,^46^), the strength of these estimates is limited by a focus on isolated RPE rather than intact retinas,^45^ and critically, a lack of data in multiple species.

Our data suggested that for soluble materials with a radius of 1 to 45 nm in rabbits, size is a strong predictor of vitreal PK, while charge and hydrophobicity, at least within the ranges tested here, do not measurably affect vitreal clearance. Increasing the hydrodynamic size of a therapeutic agent appears to be part of a viable strategy to reduce dosing frequency. Whether and to what extent retinal distribution and therapeutic efficacy may be affected by manipulation of charge, hydrophobicity, and molecular size remains to be seen.

## Supplementary Material

Supplement 1

Supplement 2
