# Supplementary material for: Influence of Charge, Hydrophobicity, and Size on Vitreous Pharmacokinetics of Large Molecules
Source: Transl Vis Sci Technol. 2019 Nov 1;8(6):1. doi: 10.1167/tvst.8.6.1 (PMC6827426; doi:10.1167/tvst.8.6.1)
Supplement: Supplement 1 [file tvst-08-05-21_s01.pdf]

Supplemental Material

Table S1. <separate file>

Table S2. Interstudy variability in ranibizumab vitreal PK in New Zealand White rabbits following ITV administration.

Parameter	Units	Average value	S.D.	Range (n=13 studies)
**AUC_inf/dose**	ug/mL*day	3610	1370	2221 - 7760
**Half-life**	days	3.4	0.71	2.4 – 5.5
**CL**	mL/day	0.30	0.08	0.13 – 0.45
**Vss**	mL	1.5	0.5	0.54 – 2.35

Table S3. Determination of zeta potential from laser Doppler velocimetry measurements on a Zetasizer Nano (Malvern instruments).

Sample	Calculated Charge at pH 7.4	Zeta Potential (mV)
**Rabbit Albumin**	-19	-11.0 ± 1.0; -14.2 ± 1.1
**Lampalizumab**	-1.8	-5.8 ± 0.42
**Ranibizumab**	+2.3	*0.003 ± 1.24*
**Anti-LTα_WT**	+16.9	4.5 ± 0.5
Anti-LTα_+3	*+22.9*	*6.1 ± 1.1*

Black font: 10 mg/mL protein in 20 mM sodium phosphate, 20 mM NaCL, pH 7.4
Blue font: 10 mg/mL protein in 10 mM Tris-HCl, 10 mM NaCL, pH 7.4
Note: Values in *italics* did not pass quality control of zetasizer software; determining zeta potential from light scattering measurements for smaller particle sizes, and in the presence of salt needed for solubility of some molecules at pH 7.4, is challenging (Malvern Instruments Technical Note, 2017).

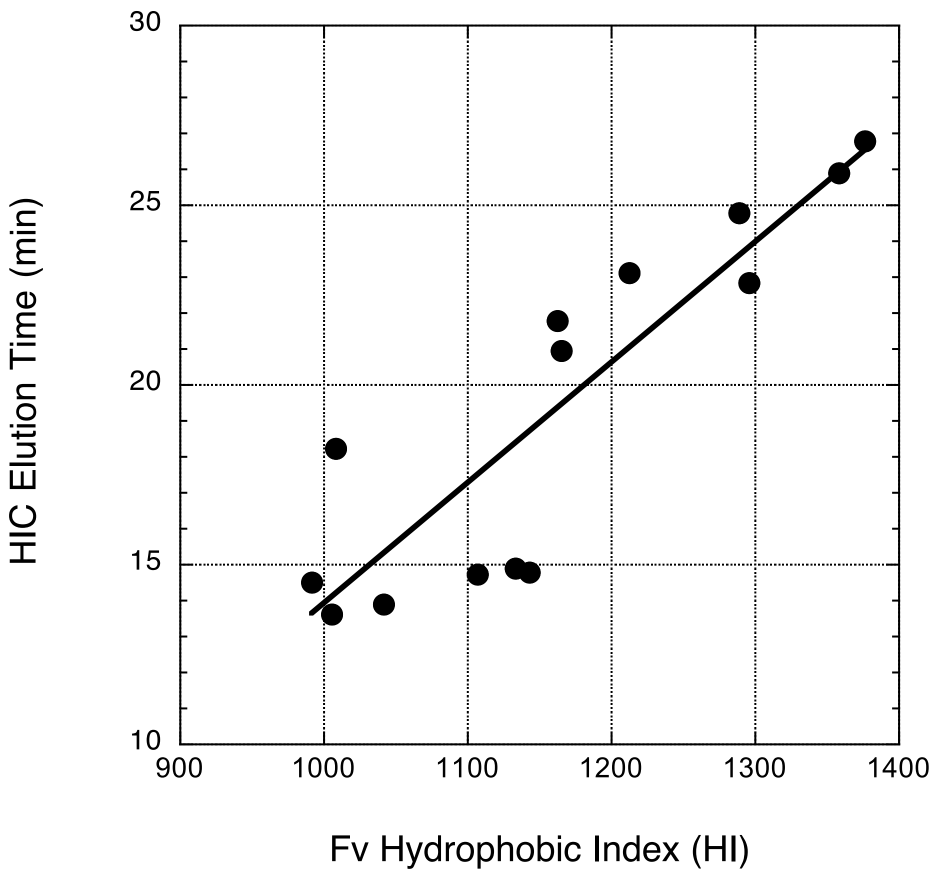

Figure S1. Correlation of measured elution time on a hydrophobic interaction chromatography column (HIC) to hydrophobicity index of the variable domain unit (FvHI) calculated from the amino acid sequence. A collection of 14 antibody Fab fragments were used to demonstrate this correlation. A linear regression analysis (solid line) on these data indicates R^2=0.8826.

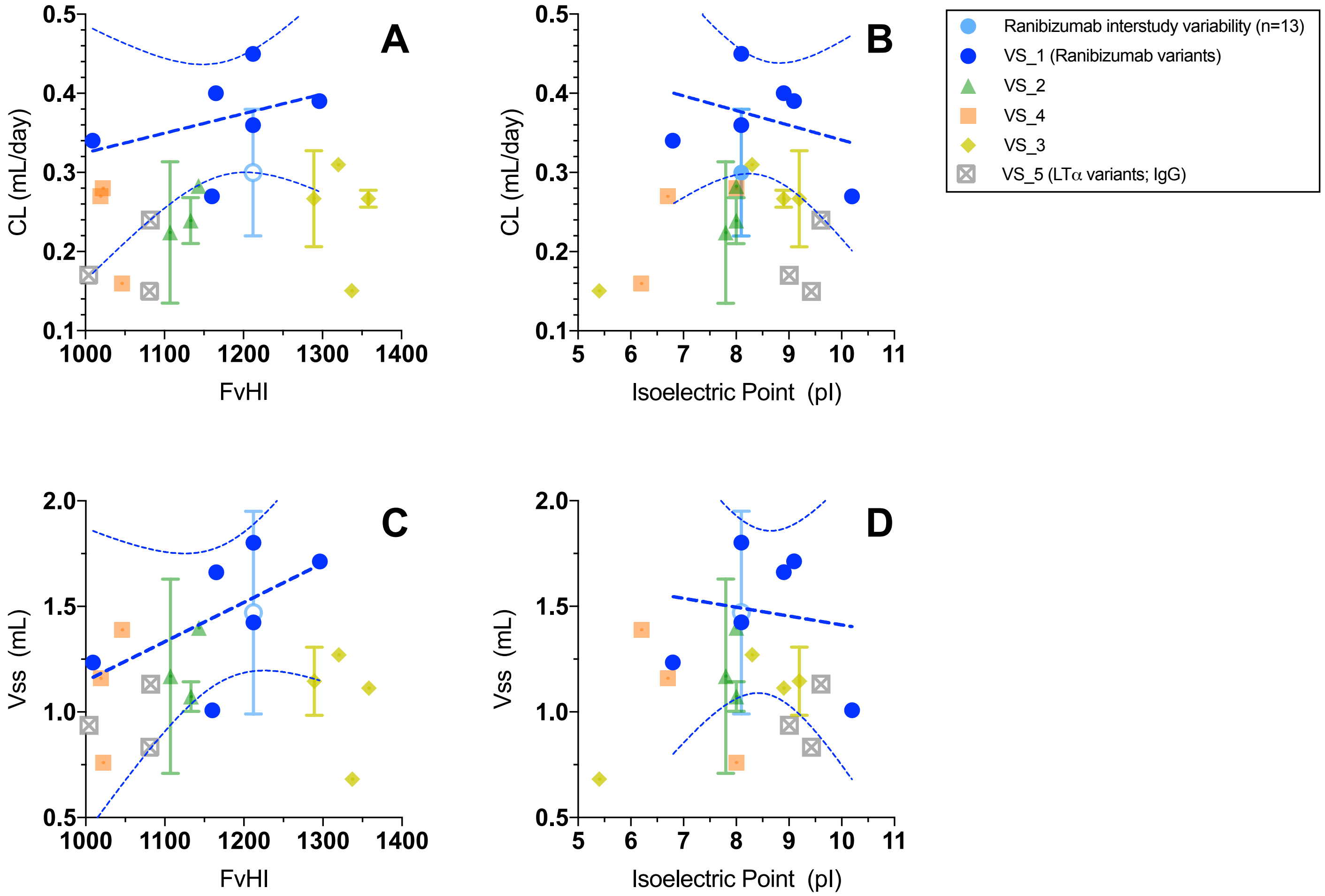

Figure S2. Relationships between vitreous PK (Clearance, CL; Volume of distribution, Vss) and molecule hydrophobicity (FvHI) or charge (pI) in New Zealand White rabbit. Linear regressions with 95% confidence intervals are shown in dark blue for ranibizumab series, with R^2 = 0.1483 (A), R^2 = 0.1218 (B), R^2 = 0.3280 (C), and R^2 = 0.02432 (D). Note that standard deviation across 13 studies of ITV ranibizumab, shown in light blue, exceeds the magnitude of distribution for any variant series shown.

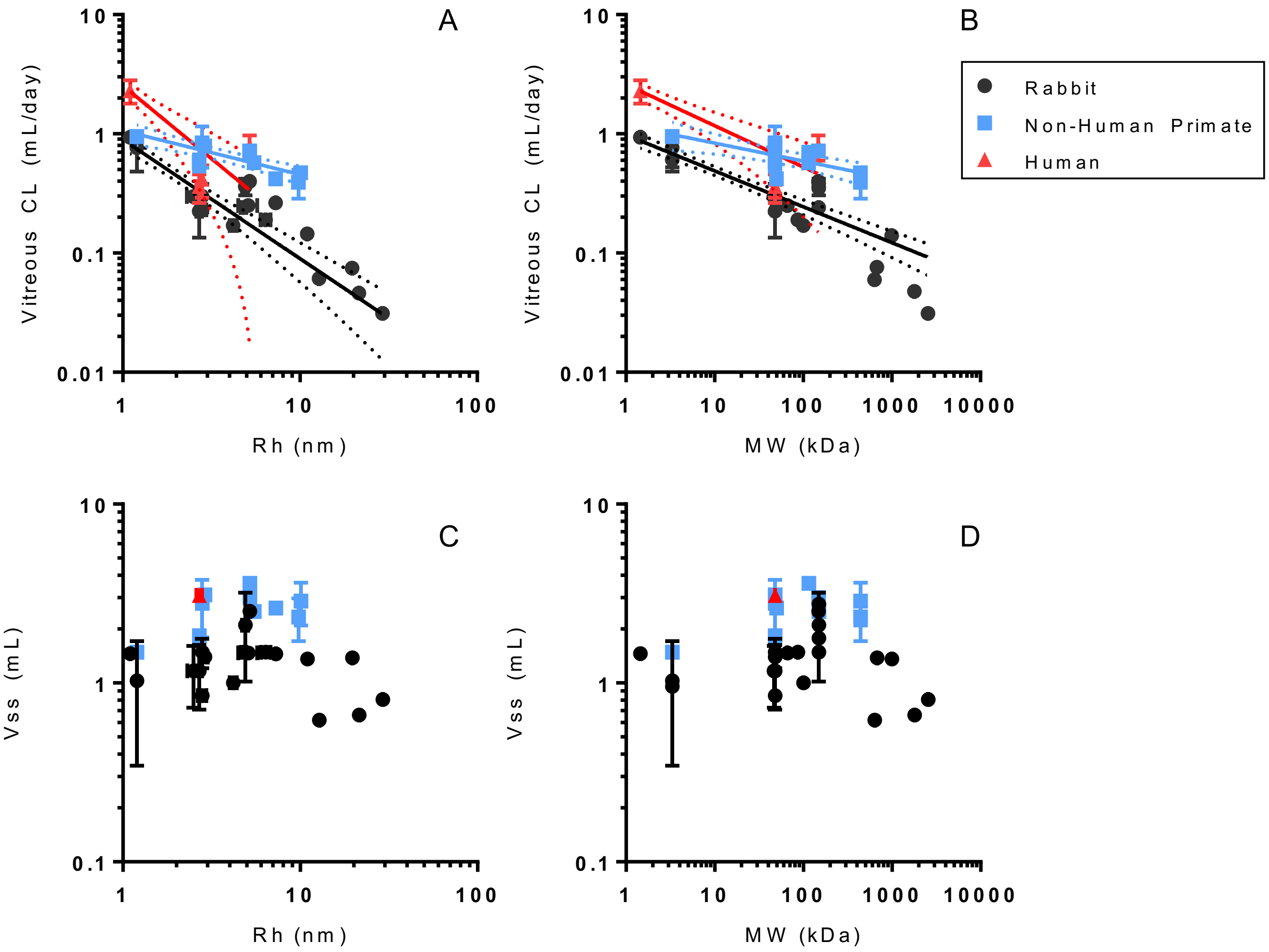

Figure S3. Relationships between vitreous PK (Clearance, CL; Volume of distribution, Vss) and molecular size (hydrodynamic radius, R_H; molecular weight, MW) in New Zealand White rabbits (black), non-human primates (NHP, blue), and humans (red). Log-log regressions with 95% confidence intervals are shown for CL and R_H (A, rabbit R^2 = 0.7659, NHP R^2 = 0.7296, human R^2 = 0.7681) and MW (B, rabbit R^2 = 0.7612, NHP R^2 = 0.5720, human R^2 = 0.8134).

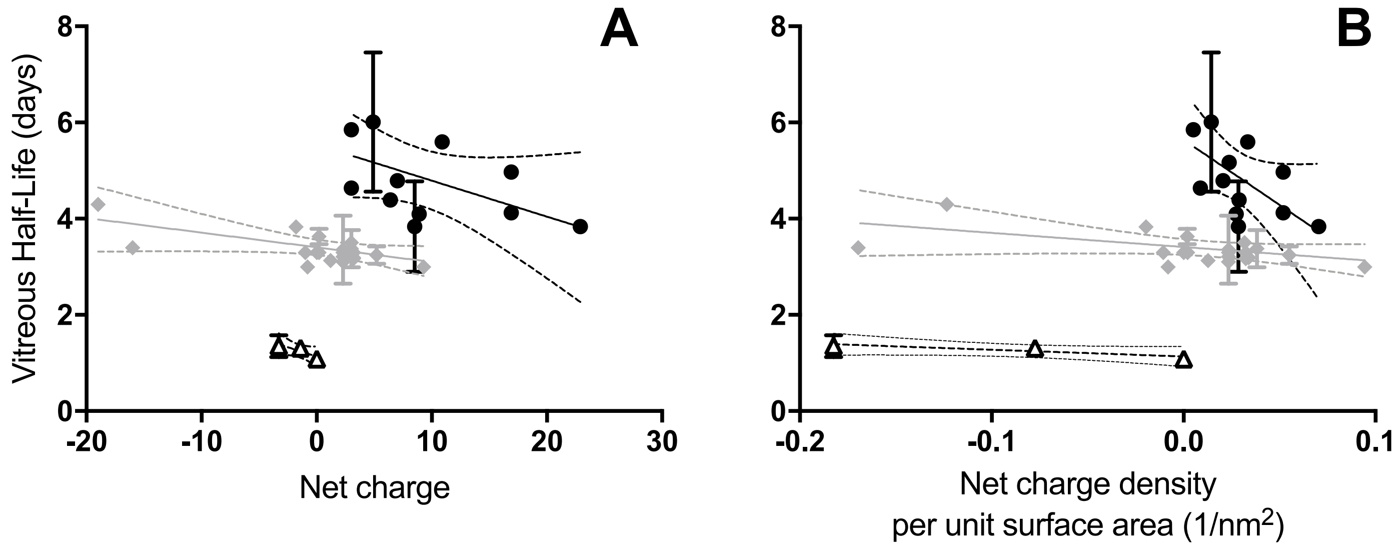

Figure S4. Relationships between vitreous half-life and net charge (A) or net charge density per unit surface area (1/nm2, B) in New Zealand White rabbits for molecules of approximately 5 (△), 50 (◆), or 150 (●) kDa molecular weight. Surface areas are based on hydrodynamic radius as reported in Table S1. Linear regressions with 95% confidence intervals (lines) for each molecular weight class had R^2 = 0.4191 (5 kDa), 0.1689 (50 kDa), and 0.09445 (150 kDa) for net charge, and R^2 = 0.0.4191 (5 kDa), 0.07072 (50 kDa), and 0.2171 (150 kDa) for net charge density.
