# Supplementary material for: Influence of Charge, Hydrophobicity, and Size on Vitreous Pharmacokinetics of Large Molecules
Source: Transl Vis Sci Technol. 2019 Nov 1;8(6):1. doi: 10.1167/tvst.8.6.1 (PMC6827426; doi:10.1167/tvst.8.6.1)
Supplement: Supplement 2 [file tvst-08-05-21_s02.pdf]

Table S1. Molecule information and key data included in evaluation of correlation between charge, hydrophobicity, molecular size, and vitreal pharmacokinetics.

Molecule ID	Variant Series	Molecule Name	Molecule Format	pI	Net Charge (pH 7.4)	FvHI	HIC Elution (min)	Molecular Weight (kDA)	Rh (nm)	Rabbit Half-Life (days)	n	Source	NHP Half-Life (days)	n	Source	Human Half-Life (days)	n	Source	Detection method(s)[a]
TA_1	VS_1	Ranibizumab	Fab	8.1	2.3	1212	23.134	48	2.8 ± 0.11	3.36 ± 0.71	13	[1, 2]	2.32 ± 0.055	3	[1, 3, 4]	6.7 ± 2.0	2	[1]	ELISA; gamma
TA_2	VS_1	RBZ_v+7	Fab	10.2	9.3	1009	18.198	48	--	3	1	[1]	--	--	--	--	--	--	ELISA
TA_3	VS_1	RBZ_v-3	Fab	6.8	-0.8	1163	--	48	--	3	1	[1]	--	--	--	--	--	--	ELISA
TA_4	VS_1	RBZ_HI low	Fab	8.9	2.3	1165	20.974	48	--	3.3	1	[1]	--	--	--	--	--	--	ELISA
TA_5	VS_1	RBZ_HI high	Fab	9.1	3.2	1296	22.846	48	--	3.3	1	[1]	--	--	--	--	--	--	ELISA
TA_6	VS_2	Lampalizumab	Fab	7.8	-1.8	1107	--	48	2.7 ± 0.1	3.83 ± 0.01	2	[1, 5]	2.38 ± 0.035	2	[6]	5.9 ± 1.59	2	[7]	ELISA
TA_7	VS_2	AfD.v8	Fab	8	0.2	1133	14.877	48	--	3.3	1	[1, 5]	--	--	--	--	--	--	ELISA
TA_8	VS_2	AfD.v14	Fab	8	0.2	1143	14.804	48	2.9 ± 0.11	3.6	1	[1, 5]	2.72	1	[1]	--	--	--	ELISA
TA_9	VS_3	TA_9	Fab	8.9	3.2	1358	25.85	48	--	3.2	1	[1]	--	--	--	--	--	--	ELISA
TA_10	VS_3	TA_10	Fab	9.2	5.2	1289	24.802	48	--	3.2	1	[1]	--	--	--	--	--	--	ELISA
TA_11	VS_3	TA_11	Fab	5.4	-16	1337	--	48	--	3.4	1	[1]	--	--	--	--	--	--	ELISA
TA_12	VS_3	TA_12	Fab	8.3	1.2	1320	--	48	--	3.1	1	[1]	--	--	--	--	--	--	ELISA
TA_13	VS_4	5B3_WT	Fab	8	3	1022	20.745	48	--	3.5	1	[1]	--	--	--	--	--	--	ELISA
TA_14	VS_4	5B3_v1	Fab	6.7	0	1019	21.383	48	--	3.3	1	[1]	--	--	--	--	--	--	ELISA
TA_15	VS_4	5B3_v2	Fab	6.2	-1	1046	25.435	48	--	3.3	1	[1]	--	--	--	--	--	--	ELISA
TA_16	VS_5	LTα_WT	IgG	9.43	16.9	1081	--	150	--	4.1	1	[1]	--	--	--	--	--	--	ELISA
TA_17	VS_5	LTα_v-4	IgG	9.01	8.9	1004	--	150	--	4.1	1	[1]	--	--	--	--	--	--	ELISA
TA_18	VS_5	LTα _v+3	IgG	9.61	22.9	1082	--	150	--	3.8	1	[1]	--	--	--	--	--	--	ELISA
TA_19	--	Vancomycin	Peptide	--	--	--	--	1.45	1.1	1.08 ± 0.06	2	[8]	--	--	--	1.55 ± 0.68	3	[9, 10]	--
TA_20	--	--	peptide	--	--	--	--	3.318	2.09	1.31 ± 0.079	3	[1]	--	--	--	--	--	--	LCMS
TA_21	--	--	peptide	--	--	--	--	3.317	2.18	1.35 ± 0.23	2	[1]	--	--	--	--	--	--	LCMS; gamma
TA_22	--	Brolucizumab	Single chain FV	6.5[b]	-3.9	1393	--	26.31	--	--	--	--	2.37	1	[11]	--	--	--	--
TA_23	--	RabFab	Fab	7.8	3	--	--	48	2.5 ± 0.2	3.3 ± 0.48	4	[1, 12, 13]	--	--	--	--	--	--	ELISA; LCMS
TA_24	--	anti-gD fab	Fab	9.1	3.2	--	--	48	2.8 ± 0.16	3.17	1	[1, 14]	--	--	--	--	--	--	ELISA
TA_25	--	Albumin	Protein	5.6[c]	-19	--	--	66.5	3.5	4.3	1	[15]	--	--	--	--	--	--	--
TA_26	--	Aflibercept	Fc-Fusion	9.2	7	--	--	97	5.2	4.79	1	[16]	3.57	1	[1]	--	--	--	ELISA
TA_27	--	anti-gD F(ab')2	F(ab')2	9.1	6.4	--	--	100	4.2 ± 0.19	4.39	1	[14]	--	--	--	--	--	--	ELISA
TA_28	--	Bevacizumab	IgG	8.8	4.9	--	--	149	5.2	6.01 ± 1.45	3	[17-20]	2.9	1	[1]	9.15 ± 2.0	5	[8, 21-24]	ELISA
TA_29	--	Rabbit IgG	IgG	9.2	8.5	--	--	150	4.86 ± 0.16	3.84 ± 0.94	2	[1]	--	--	--	--	--	--	ELISA
TA_30	--	anti-gD IgG	IgG	9	6.9	--	--	150	4.8 ± 0.16	5.17	1	[14]	--	--	--	--	--	--	ELISA
TA_31	--	Rituximab	IgG	9.4	16.9	--	--	150	--	4.97	1	[17, 25]	--	--	--	--	--	--	ELISA
TA_32	--	rhuMabHER2	IgG	9.2	10.9	--	--	150	--	5.6	1	[26]	--	--	--	--	--	--	ELISA
TA_33	--	VA2	CrossMab bispecific	--	--	--	--	150	5.5	--	--	--	3.02	1	[27]	--	--	--	--
TA_34	--	500 kDa HA	Polymer	--	-953	--	--	500	45	29.5	1	[28]	--	--	--	--	--	--	--
TA_35	--	18kDa HA	Polymer	--	-46	--	--	18	--	4.4	1	[28]	--	--	--	--	--	--	--
TA_36	--	FITC-dextran_10.5	Polysaccharide	--	--	--	--	10.5	--	2.89	1	[29]	--	--	--	--	--	--	--
TA_37	--	FITC-dextran_67	Polysaccharide	--	--	--	--	67	--	5.78	1	[29]	--	--	--	--	--	--	--

TA_38	--	FITC-dextran_157	Polysaccharide	--	--	--	--	157	--	6.93	1	[29]	--	--	--	--	--	--	--
TA_39	--	Abicipar pegol	Protein-polymer conjugate	--	--	--	--	34	--	6	1	[30, 31]	--	--	--	13.4		[30, 31]	--
TA_40	--	Pegaptanib	Protein-polymer conjugate	--	--	--	--	50	7.3	3.82	1	[17, 32]	3.92	1	[33]	8.2 ± 1.7	3	[34-36]	--
TA_41	--	RabFab-20 kDA PEG	Protein-polymer conjugate	7.8	3	--	--	66.5	5.2 ± 0.3	4.64	1	[12]	--	--	--	--	--	--	ELISA
TA_42	--	RabFab-40 kDa PEG	Protein-polymer conjugate	7.8	3	--	--	86.5	6.9 ± 0.3	5.85	1	[12]	--	--	--	--	--	--	ELISA
TA_43	--	AfD.v3 Octamer	Protein-polymer conjugate	8	0.2	--	--	440	10.1	--	--	--	4.22 ± 1.09	2	[1]	--	--	--	ELISA
TA_44	--	AfD.v3.2 Octamer	Protein-polymer conjugate	9.4	5.2	--	--	440	9.8	--	--	--	4.45 ± 0.28	3	[1]	--	--	--	Gamma
TA_45	--	TA_45	Protein-polymer conjugate	7.8	3	--	--	1000	11	7.8	1	[1]	--	--	--	--	--	--	ELISA
TA_46	--	HA150K-rabFab	Protein-polymer conjugate	--	-329	--	--	679	17.3	11.9	1	[13]	--	--	--	--	--	--	Gamma
TA_47	--	HA40K-rabFab	Protein-polymer conjugate	--	-57	--	--	636.2	12.8	7.6	1	[13]	--	--	--	--	--	--	ELISA; LCMS
TA_48	--	HA200K-rabFab	Protein-polymer conjugate	--	-392	--	--	1805.3	21.5	10.2	1	[13]	--	--	--	--	--	--	ELISA; LCMS
TA_49	--	HA600K-rabFab	Protein-polymer conjugate	--	-1324	--	--	2569.2	29.2	18.3	1	[13]	--	--	--	--	--	--	ELISA; LCMS
TA_50	--	--	Protein-polymer conjugate	--	--	--	--	1150	24.3	--	--	--	10	1	[1]	--	--	--	ELISA
TA_51	--	--	Protein-polymer conjugate	--	--	--	--	570	19.1	--	--	--	6	1	[1]	--	--	--	ELISA

pI: isoelectric point; FvHI: hydrophobicity of the antibody Fv domain; HIC: hydrophobicity interaction column; Rh: hydrodynamic radius; NHP: nonhuman primate; --: not assessed or reported

[a] Methods are for internally conducted studies only

[b] pI calculated for pseudo Fab

[c] pI and charge calculated for rabbit SA

References

1. *Internal Studies*.
2. Gaudreault, J., et al., *Pharmacokinetics and retinal distribution of ranibizumab, a humanized antibody fragment directed against VEGF-A, following intravitreal administration in rabbits.* Retina, 2007. **27**(9): p. 1260-6.
3. Gaudreault, J., et al., *Preclinical pharmacokinetics of Ranibizumab (rhuFabV2) after a single intravitreal administration.* Investigative ophthalmology & visual science, 2005. **46**(2): p. 726-733.
4. Mordenti, J., et al., *Comparisons of the intraocular tissue distribution, pharmacokinetics, and safety of 125I-labeled full-length and Fab antibodies in rhesus monkeys following intravitreal administration.* Toxicol Pathol, 1999. **27**(5): p. 536-44.
5. Tesar, D., et al., *Protein engineering to increase the potential of a therapeutic antibody Fab for long-acting delivery to the eye AU - Tesar, Devin.* mAbs, 2017. **9**(8): p. 1297-1305.
6. Le, K.N., et al., *A mechanistic pharmacokinetic/pharmacodynamic model of factor D inhibition in cynomolgus monkeys by lampalizumab for the treatment of geographic atrophy.* J Pharmacol Exp Ther, 2015. **355**(2): p. 288-96.
7. Le, K.N., et al., *Population Pharmacokinetics and Pharmacodynamics of Lampalizumab Administered Intravitreally to Patients With Geographic Atrophy.* CPT Pharmacometrics Syst Pharmacol, 2015. **4**(10): p. 595-604.

8. del Amo, E.M., et al., *Intravitreal clearance and volume of distribution of compounds in rabbits: In silico prediction and pharmacokinetic simulations for drug development.* Eur J Pharm Biopharm, 2015. **95**(Pt B): p. 215-26.
9. Waite, K.E. and P.R. Pavan, *Half-life of Intravitreal Vancomycin in Humans.* Invest Ophthalmol Vis Sci, 2003. **44**(13): p. 1859.
10. Radhika, M., et al., *Pharmacokinetics of intravitreal antibiotics in endophthalmitis.* J Ophthalmic Inflamm Infect, 2014. **4**: p. 22.
11. Nimz, E.L., et al., *Intraocular and systemic pharmacokinetics of brolucizumab (RTH258) in nonhuman primates*, in *ARVO (Association for Research in Vision & Ophthalmology) Annual Meeting*. 2016: Seattle, WA.
12. Shatz, W., et al., *Contribution of Antibody Hydrodynamic Size to Vitreal Clearance Revealed through Rabbit Studies Using a Species-Matched Fab.* Mol Pharm, 2016. **13**(9): p. 2996-3003.
13. Famili, A., et al., *Hyaluronic Acid-Antibody Fragment Bioconjugates for Extended Ocular Pharmacokinetics.* Submitted, 2019
14. Gadkar, K., et al., *Design and Pharmacokinetic Characterization of Novel Antibody Formats for Ocular Therapeutics.* Invest Ophthalmol Vis Sci, 2015. **56**(9): p. 5390-400.
15. Maurice, D.M., *Protein dynamics in the eye studied with labelled proteins.* Am J Ophthalmol, 1959. **47**(1 Pt 2): p. 361-8.
16. *Assessment report for aflibercept (EMA/646256/2012)*, E.M.A.C.f.M.P.f.H. Use, Editor. 2012, EMA (European Medicines Agency). p. 18.
17. Del Amo, E.M. and A. Urtti, *Rabbit as an animal model for intravitreal pharmacokinetics: Clinical predictability and quality of the published data.* Exp Eye Res, 2015. **137**: p. 111-24.
18. Bakri, S.J., et al., *Pharmacokinetics of intravitreal bevacizumab (Avastin).* Ophthalmology, 2007. **114**(5): p. 855-9.
19. Ahn, J., et al., *Pharmacokinetics of intravitreally injected bevacizumab in vitrectomized eyes.* J Ocul Pharmacol Ther, 2013. **29**(7): p. 612-8.
20. Sinapis, C.I., et al., *Pharmacokinetics of intravitreal bevacizumab (Avastin(R)) in rabbits.* Clin Ophthalmol, 2011. **5**: p. 697-704.
21. Csaky, K.G., et al., *Pharmacokinetics of Intravitreal Bevacizumab in Humans.* Invest Ophthalmol Vis Sci, 2007. **48**(13): p. 4936.
22. Zhu, Q., et al., *Vitreous levels of bevacizumab and vascular endothelial growth factor-A in patients with choroidal neovascularization.* Ophthalmology, 2008. **115**(10): p. 1750-5, 1755.e1.
23. Krohne, T.U., et al., *Intraocular pharmacokinetics of bevacizumab after a single intravitreal injection in humans.* Am J Ophthalmol, 2008. **146**(4): p. 508-12.
24. Meyer, C.H., T.U. Krohne, and F.G. Holz, *Intraocular pharmacokinetics after a single intravitreal injection of 1.5 mg versus 3.0 mg of bevacizumab in humans.* Retina, 2011. **31**(9): p. 1877-84.
25. Kim, H., et al., *The pharmacokinetics of rituximab following an intravitreal injection.* Exp Eye Res, 2006. **82**(5): p. 760-6.
26. Mordenti, J., et al., *Intraocular pharmacokinetics and safety of a humanized monoclonal antibody in rabbits after intravitreal administration of a solution or a PLGA microsphere formulation.* Toxicological Sciences, 1999. **52**(1): p. 101-106.
27. Regula, J.T., et al., *Targeting key angiogenic pathways with a bispecific CrossMAb optimized for neovascular eye diseases.* EMBO Mol Med, 2016. **8**(11): p. 1265-1288.
28. Laurent, U.B. and J.R. Fraser, *Turnover of hyaluronate in the aqueous humour and vitreous body of the rabbit.* Exp Eye Res, 1983. **36**(4): p. 493-503.
29. Johnson, F. and D. Maurice, *A simple method of measuring aqueous humor flow with intravitreal fluoresceinated dextrans.* Experimental Eye Research, 1984. **39**(6): p. 791-805.
30. Stewart, M.W., *Extended Duration Vascular Endothelial Growth Factor Inhibition in the Eye: Failures, Successes, and Future Possibilities.* Pharmaceutics, 2018. **10**(1).
31. Campochiaro, P.A., et al., *Treatment of diabetic macular edema with a designed ankyrin repeat protein that binds vascular endothelial growth factor: a phase I/II study.* Am J Ophthalmol, 2013. **155**(4): p. 697-704, 704.e1-2.
32. Group, E.S., *Preclinical and phase 1A clinical evaluation of an anti-VEGF pegylated aptamer (EYE001) for the treatment of exudative age-related macular degeneration.* Retina, 2002. **22**(2): p. 143-152.

33. Drolet, D.W., et al., *Pharmacokinetics and safety of an anti-vascular endothelial growth factor aptamer (NX1838) following injection into the vitreous humor of rhesus monkeys.* Pharm Res, 2000. **17**(12): p. 1503-10.
34. Basile, A.S., et al., *Population pharmacokinetics of pegaptanib sodium (Macugen((R))) in patients with diabetic macular edema.* Clin Ophthalmol, 2015. **9**: p. 323-35.
35. Group, M.S., *Pegaptanib 1-Year Systemic Safety Results from a Safety–Pharmacokinetic Trial in Patients with Neovascular Age-Related Macular Degeneration.* Ophthalmology, 2007. **114**(9): p. 1702-1712.e2.
36. Basile, A.S., et al., *Population pharmacokinetics of pegaptanib in patients with neovascular, age-related macular degeneration.* J Clin Pharmacol, 2012. **52**(8): p. 1186-99.
